# Acute Left Atrial Thrombus Formation on Resected Residual Cribriform Septum after Atrial Septal Defect Surgery

**Published:** 2015-04-03

**Authors:** Naser Hemati, Alireza Poormotaabed, Samsam Dabiri, Feridoun Sabzi

**Affiliations:** Imam Ali Hospital, Kermanshah University of Medical Sciences, Kermanshah, Iran.

**Keywords:** Heart septal defects, atrial, Cardiac surgical procedures, Thrombosis, Heart septum

## Abstract

Acute left atrial thrombosis at the site of the resection of the primary cribriform septum is an exceedingly rare and important complication after atrial septal defect (ASD) closure with a pericardial or synthetic patch. This case report presents a mobile thrombus noted on the left atrium at the raw surface site of a resected cribriform primary septum that was not caught in the suture line with the pericardial patch for the closure of the ASD in a 30-year-old woman with an uncomplicated ASD surgery. The patient had no symptoms in the postoperative period, and routine postoperative transesophageal echocardiography revealed a large pedunculated and mobile mass (thrombosis) at the left atrial side of the interatrial septum at the level of the implanted pericardial patch. The thrombus was successfully treated with surgery. The patient had an uneventful recovery in the postoperative period and was discharged from the hospital 15 days after admission. One-year follow-up showed no evidence of clot recurrence in the left or right atrium**.**

## Introduction

The surgical repair of the atrial septal defect (ASD) is a well-established procedure and is very safe, with a negligible mortality rate.^1^ Chessa et al.^2^ reported that both device and surgical patch repairs of the adult ASD might result in acute left and right atrial thrombosis formation.^2^ However, the formation of an acute thrombus after the pericardial patch repair of the ASD at the site of the cribriform ridge is exceedingly rare, and there are limited data detailing such a complication.^2^ Galal et al.^3^ classified complications after the ASD closure as mild, moderate, or severe. Mild complications included small pericardial effusions, headaches, first-degree atrioventricular (AV) block, and atrial rhythm disturbances; moderate complications comprised pneumonia and atelectasis, paroxysmal supraventricular tachycardia, and AV junctional rhythm; and severe complications consisted of bleeding that required reoperation and transient neurologic events. (Because of rarity, left or right atrial thrombosis was not considered a major complication.) The early natural history of the ASD was first described by Rodriguez,^4^ who suggested that there was significant morbidity and mortality associated with unrepaired defects, so most patients with evidence of an important left-to-right shunt were referred for closure.

The surgical repair of the ASD began before the development of cardiopulmonary bypass (CPB), and in 1953 it became the first intracardiac defect successfully corrected under CPB. Transcatheter closure was first reported by King et al.^5^ in 1976, and it has evolved substantially since then. The surgical closure of the ASD remains the gold standard, with mortality rates approaching zero.^5^ The pericardial closure of the ASD is a common and routine procedure. Of the reported complications of this technique, thrombus formation, particularly on the residual resected cribriform septum for pericardial patch repair, is exceedingly rare. Reports of important thrombotic complications are rare, and anticoagulation during the procedure and after surgery is, therefore, not routine. Consequently, there is little experience with the postoperative management of left atrial thrombosis after the ASD patch repair. In some cases, the suture line serves as the nidus for thrombus formation, and patients tend to present with transient ischemic events almost immediately after surgery.

The present case is unique in that not only was the patient asymptomatic when the atrial thrombus was fortuitously discovered but also thrombosis formation had occurred at the ridge of the resected residual cribriform plate. This raw surface exposed a nidus for the activation of coagulation cascade. Moreover, the case underlines the importance of the renewal of anticoagulation therapy in case of the resection of the primary cribriform plate for pericardial patch repair.

## Case Report

**Figure 1:**
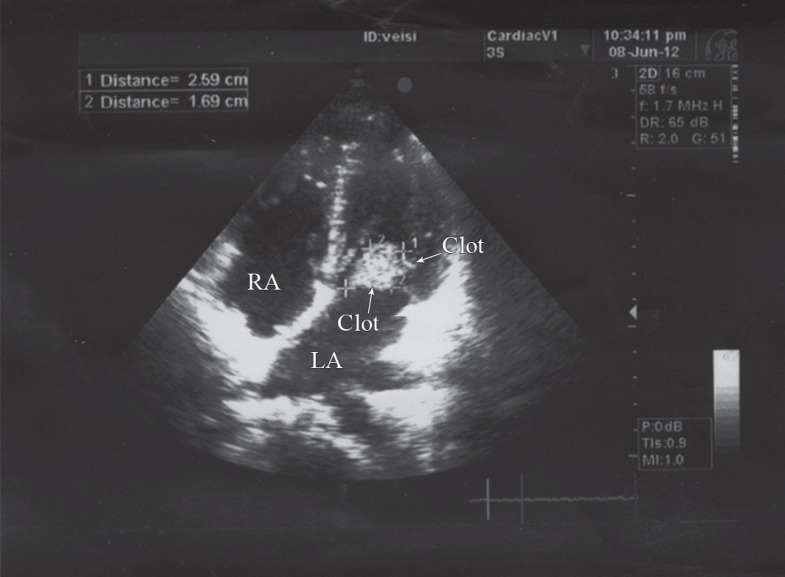
Transthoracic echocardiography in the four-chamber view, showing a large clot in the left atrium, attached via a long stalk to the residual resected interatrial septum (arrows)

RA, Right atrium; LA, Left atrium

**Figure 2:**
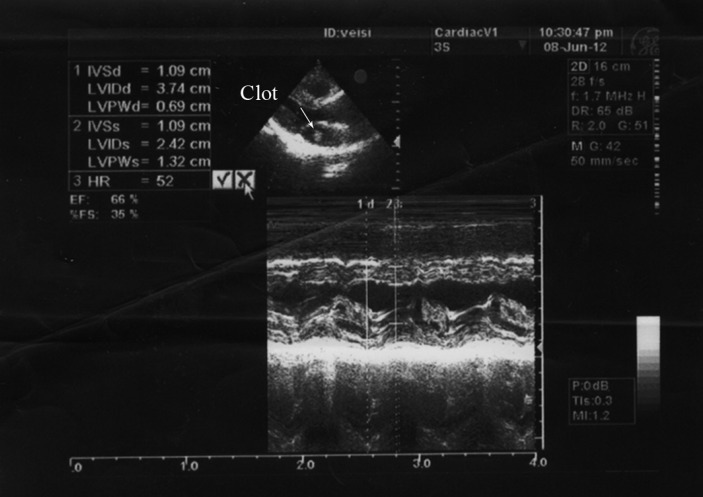
Transthoracic echocardiography in M mode and parasternal view, showing a clot (arrow) attached to the residual resected interatrial septum. No pericardial effusion is seen

A 30-year-old woman was referred to our center one week after an open heart surgery for an ASD closure with a pericardial patch. Routine postoperative transesophageal echocardiography illustrated a large pedunculated and mobile mass (thrombosis) at the left atrial side of the interatrial septum at the level of the implanted pericardial patch (Figures 1 and 2). The patient's rhythm was normal sinus rhythm, and there were no factors predictive of atrial fibrillation.^6^ Extensive laboratory investigation as regards thrombophilic disorders revealed no pathologic values.

After providing an informed consent, the patient was transferred to the operating room and placed on the operating room table in supine position. Upon the induction of general endotracheal anesthesia and placement of indwelling arterial and venous monitoring lines, the patient was prepped and draped in the usual sterile fashion from chin to groins, and a full midline vertical skin incision was performed in the sternum. The dissection was carried through the deeper planes until the sternum was scored and divided. With an oscillating saw, a small portion of the anterior pericardium was procured for the patch closure of the segment of the ASD during the procedure. Purse strings were deployed on the ascending aorta on the right and the atrial appendage. After systemic heparinization, central aortobicaval cannulation was done for CPB. Both caval veins were encircled with surgical tapes. After mild hypothermic CPB and cross-clamping of the aorta, intermittent cold cardioplegic infusion was administrated in an antegrade fashion into the aortic root. Through a standard right atriotomy, the old pericardial patch was opened and the left atrium explored for thrombosis. The thrombosis was attached via a stalk to the raw surface of the interatrial septum underneath the suture line at the site of the resection of the residual cribriform of the primary septum. The old pericardial patch and thrombosis were removed, and the ASD was closed with a new pericardial patch. De-airing was performed through the aortic root and cardiac apex before aortic declamping. The right atriotomy was closed in two layers with running 4-0 Viline sutures. Venous decannulation was followed by aortic decannulation and administration of protamine sulfate. All the cannulation sites were oversewn with 4-0 Viline sutures, and the cannulation sites were hemostatic. With the patient having good hemodynamics and hemostasis, the sternum was then closed with stainless steel wires. The subcutaneous tissues were closed in layers with reabsorbable monofilament sutures. The patient was transferred in very stable condition to the adult intensive care unit.

## Discussion

The case shows the importance of considering the possibility of thrombus formation at the raw surface of the resected cribriform plate, early after the patch repair of the ASD with an autologous pericardium or synthetic patch. It should also be considered that the majority of data evaluating the treatment of atrial thrombus come from patients suffering from large embolized clot loads from deep venous thrombosis that have transferred to the right atrium. Whether these techniques can be used for postoperative thrombi is unclear. Ahmad et al.^7^ pointed out that this consideration is especially important, as the nature of a developing thrombus at a suture line might be more or less resistant to anticoagulation therapy compared with a relatively fresh, embolized thrombus caught in the atrium. This point is well illustrated in this case, whereupon intraoperative examination, the thrombus was found to be a well-formed, hard mass with a reasonable pedicle attaching it to the residual resected septum. The most significant factors associated with an increased risk of postoperative embolism are increased age of the patient, atrial fibrillation, and pulmonary hypertension.^8^^, ^^9^ The hard consistency and histologic appearance of the thrombus suggested that a response to lytic or anticoagulation therapy would be highly unlikely. One final aspect of this case involves the appropriateness of anticoagulation therapy after pericardial patch ASD repair after the resection of some part of the cribriform plate that was not caught in the suture line. For catheter-based repair of the ASD, patients are routinely anticoagulated until the endothelialization of the foreign material is believed to be complete (typically 3 months). However, in the case of primary repairs or pericardial patch repair of the patent foramen ovale or the ASD, most centers refrain from postoperative anticoagulation. Given the findings from this case and the others discussed, such anticoagulation strategies may deserve review. Furthermore, this case illustrates the importance of in-hospital echocardiography follow-up after pericardial patch repair. This approach may help to identify postoperative thrombi earlier, providing a therapeutic window for anticoagulation or lytic therapy. A combination of preoperative hypercoagulability screening, postoperative anticoagulation, and routine interval assessment by echocardiography may provide the optimal strategy. 

## Conclusion

Thrombus formation at the ridge of the residual resected septum preparing for pericardial patch ASD repair is an exceedingly rare postoperative complication that has the potential for embolic sequelae. The present case demonstrates the importance of postoperative interval imaging to detect potential thrombi prior to manifestation by an embolic event. 

## Notes:

***This paper should be cited as: ***Hemati N, Poormotaabed A, Dabiri S, Sabzi F. Acute Left Atrial Thrombus Formation on Resected Residual Cribriform Septum after Atrial Septal Defect Surgery. J Teh Univ Heart Ctr 2015;10(2):109-112.

## References

[1] Baskett RJ, Tancock E, Ross DB (2003). The gold standard for atrial septal defect closure: current surgical results, with an emphasis on morbidity. Pediatr Cardiol.

[2] Chessa M, Carminati M, Butera G, Bini RM, Drago M, Rosti L, Giamberti A, Pomè G, Bossone E, Frigiola A (2002). Early and late complications associated with transcatheter occlusion of secundum atrial septal defect. J Am Coll Cardiol.

[3] Galal MO, Wobst A, Halees Z, Hatle L, Schmaltz AA, Khougeer F, De Vol E, Fawzy ME, Abbag F, Fadley F (1994). Peri-operative complications following surgical closure of atrial septal defect type II in 232 patients--a baseline study. Eur Heart J.

[4] Rodriguez CJ, Di Tullio MR, Sacco RL, Homma S (2001). Intra-atrial thrombus after surgical closure of patent foramen ovale. J Am Soc Echocardiogr.

[5] King TD, Thompson SL, Steiner C, Mills NL (1976). Secundum atrial septal defect. Nonoperative closure during cardiac catheterization. JAMA.

[6] Sabzi F, Zokaei AH, Moloudi AR (2011). Predictors of atrial fibrillation following coronary artery bypass grafting. Clin Med Insights Cardiol.

[7] Sheikh AY, Schrepfer S, Stein W, West J, Lombard J, Burdon T, Pinsker B, Pelletier MP (2007). Right atrial mass after primary repair of an atrial septal defect: thrombus masquerading as a myxoma. Ann Thorac Surg.

[8] Hawe A, Rastelli GC, Brandenburg RO, McGoon DC (1969). Embolic complications following repair of atrial septal defects. Circulation.

[9] Dörr M, Hummel A (2007). Images in clinical medicine. Paradoxical embolism--thrombus in a patent foramen ovale. N Engl J Med.

